# Recent developments, challenges, and prospects of ultrasound-assisted oil technologies

**DOI:** 10.1016/j.ultsonch.2021.105902

**Published:** 2021-12-28

**Authors:** Idowu Adeyemi, Mahmoud Meribout, Lyes Khezzar

**Affiliations:** a Department of Electrical Engineering and Computer Science, Khalifa University of Science and Technology, P.O. Box 127788, Abu Dhabi, United Arab Emirates; b Department of Mechanical Engineering, Khalifa University of Science and Technology, P.O. Box 127788, Abu Dhabi, United Arab Emirates; c Ecole Nationale Polytechnique de Constantine, Constantine, Algeria

**Keywords:** Ultrasound, Demulsification, Emulsion, Enhanced oil recovery, HiFU

## Abstract

There has been consistent drive towards research and innovation in oil production technologies in order to achieve improved effectiveness and efficiency in their operation. This drive has resulted in breakthrough in technologies such as the application of ultrasound (US) in demulsification and enhanced oil recovery (EOR), and usage of high-volume hydraulic fracturing and special horizontal well for shale oil and gas extraction. These can be observed in the increment in the number of commercial oil technologies such as EOR projects that rose from 237 in 1996 to 375 in 2017. This sustained expansion in EOR resulted in their total oil production rising from 1.5 million barrels per day in 2005 to 2.3 million barrels per day in 2020. And this is predicted to increase to about 4.7 million barrels per day in 2040, which represent about 4% of total production. Consequently, in this review, the developments in the utilization of US either as standalone or integrated with other technologies in EOR and dehydration of water in oil emulsions were analyzed. The studies include the optimization of fluid and US properties in EOR and demulsification. Reports on the treatment of formation damage resulting from inorganic salts, organic scales, drilling fluid plugs, condensate, paraffin wax and colloidal particle with US-assisted EOR were also highlighted. Moreover, the mechanisms were examined in order to gain insightful understanding and to aid research investigations in these areas. Technologies such as US assisted green demulsification, high intensity focused ultrasound, and potential pathways in field studies were assessed for their feasibilities. It is essential to evaluate these technologies due to the significant accrued benefits in them. The usage of green demulsifiers such as deep eutectic solvents, ionic liquids and bio-demulsifiers has promising future outlook and US could enhance their technical advancement. HiFU has been applied successfully in clinical research and developments in this area can potentiality improve demulsification and interfacial studies (fluid–fluid and solid–fluid interactions). As regards field studies, there is need to increase actual well investigations because present reports have few on-site measurements with most studies being in laboratory scale. Furthermore, there is need for more detailed modeling of these technologies as it would assist in conserving resources, saving research time and fast-tracking oil production. Additional evaluative studies of conditions such as the usage of Raschig rings, crude oil salinity and high temperature which have improved demulsification of crude oil emulsions should be pursued.

## Highlights

• Progress in the application of ultrasound (US) in oil technologies was discussed.
• Mechanisms of US assisted oil technologies were examined.
• Potentiality of emerging US technologies and green solvents were assessed.
• Modeling studies in demulsification and enhanced oil recovery were highlighted.
• Effect of Raschig rings, crude oil salinity and high temperature were evaluated.

## Graphical abstract

**Figure:**
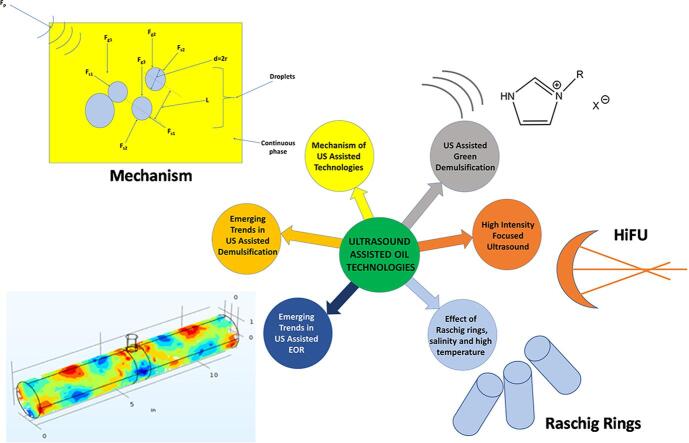

## 1 Introduction

**Fig. 1:**
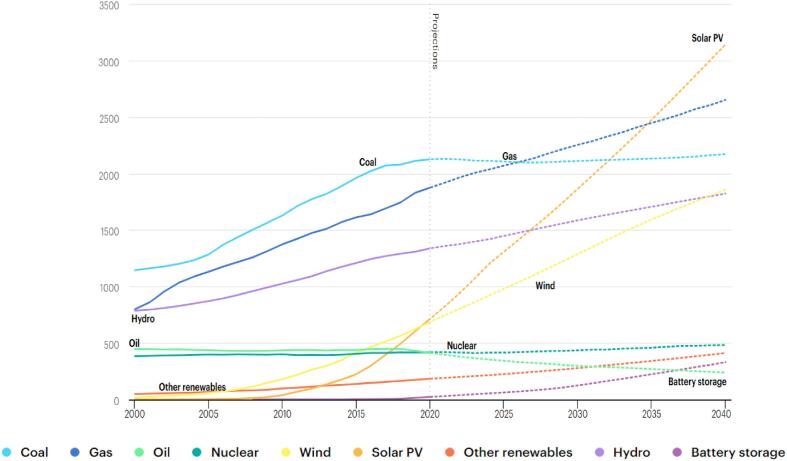
Total global installed power capacity between 2000 and 2040 in gigawatts (GW) [1].

**Fig. 2:**
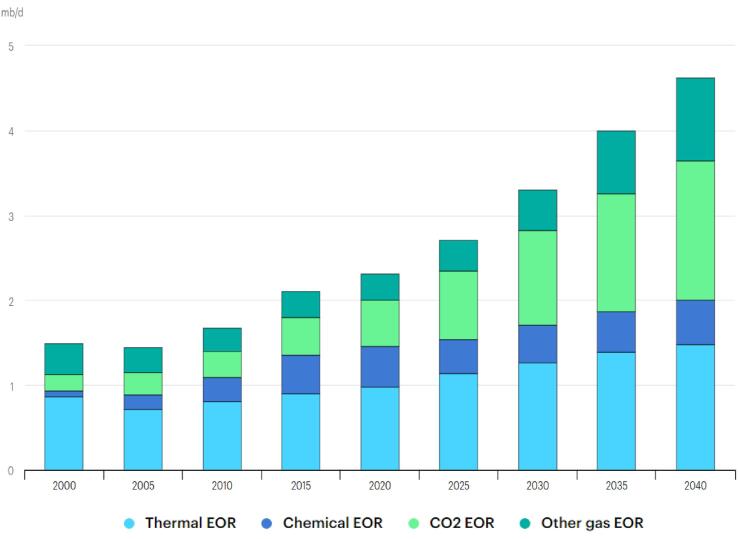
Development of EOR technologies in mb/d (million barrels per day) between 2000 and 2040 under the New Policy Scenario [4].

The total global energy demand has persistently increased for decades, and this expansion has been predicted to continue further into the future. According to the World Energy Outlook [1], the cumulative installed power capacity increased from 3890 GW in 2000 to 7800 GW in 2020 (Fig. 1). And despite the current developments in renewable energy, energy efficiency, energy conservation, and impacts of Covid-19, the energy demand continues to rise. The International Energy Agency (IEA) [2] estimated that the global energy demand would rise by 4.6% in 2021. Majority of this energy comes from fossil fuels which forms about 80% of the aggregate demand [3]. Consequently, there have been consistent drive and research towards the effective and efficient advanced oil production and exploration technologies. Developments have been witnessed in the application of ultrasound (US) in demulsification and enhanced oil recovery (EOR), high volume hydraulic fracturing and special horizontal well for shale oil and gas extraction and 3D seismic imaging. For example, the tertiary extraction of oil through EOR has witnessed significant growth over the past decades [4]. This growth can be attributed to the fact that 60–67% of crude oil remains after secondary oil recovery [43], [44], [45], [46]. Hence, in the World Energy Outlook [4], the number of commercial EOR projects increased from 237 in 1996 to 375 in 2017. This sustained expansion has resulted in their total oil production through EOR rising from 1.5 mb/d (million barrels per day) in 2005 to 2.3 mb/d in 2020. And this is predicted to increase to about 4.7 mb/d in 2040, which represent about 4% of total production (Fig. 2). Although these technologies utilize thermal, chemical and gas for the oil recovery due to their benefits, they have often faced setbacks due to challenges in their usage. Thermal EOR produces significant environmental challenges and utilizes larger fresh water supply in generation of steam. Emission of pollutants such as nitrous oxides, sulfur oxides and carbon dioxide from the combustion of gasoline (13–14.9 kg of CO_2_ gallon^-1^) or crude oil (10.5–13 kg of CO_2_ gallon^-1^) during thermal EOR is a major drawback to this technology. Moreover, the production of a unit volume of oil requires 2–10 volumes of fresh water under steam operations [5]. Similar challenge of environmental degradation occurs with chemical EOR [6], [7]. And CO_2_ EOR encounters corrosion issues, and potential pipeline and equipment leakages [8]. Hence, technologies such as ultrasound has been utilized in combination with these EOR methods in order to further boost their performance.

**Fig. 3:**
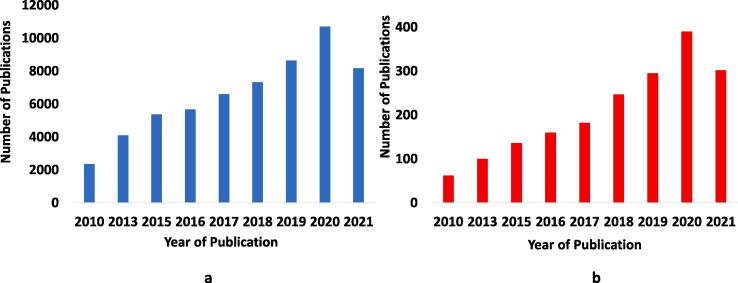
Trends in the research publications in the last decade (2010–2021): **a** EOR **b** Demulsification based on Google Scholar.

**Fig. 4:**
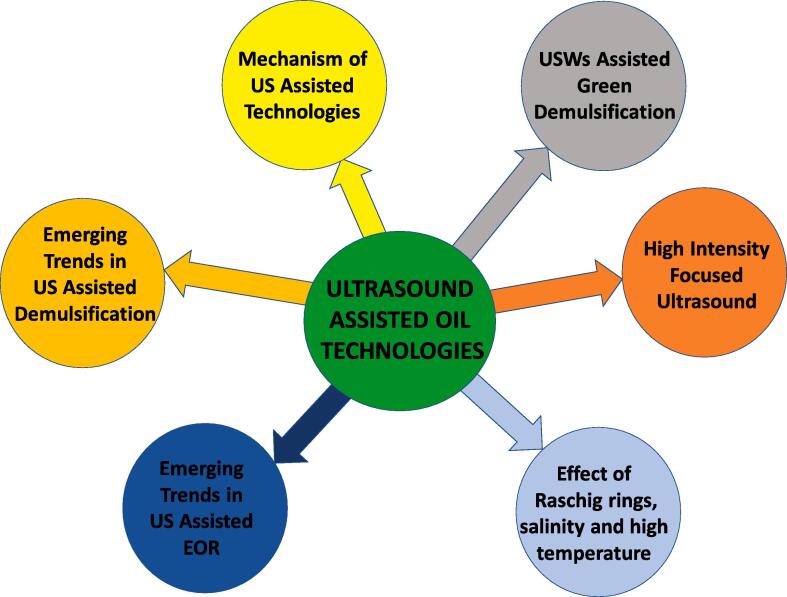
Outlook of the developments in the US assisted oil technologies.

Ultrasound assisted oil technologies have technical effectiveness, economic and environmental benefits which make them a potential solution for the challenges faced in oil production. There are several reports highlighting the improvement in oil recovery, enhancement of water-in-oil emulsions dehydration, and treatment of formation damage resulting from inorganic salts, organic scales, drilling fluid plugs, condensate, paraffin wax and colloidal particle in the past decade (Fig. 3). In a field study by Abramov and co-workers [9], the average rate of oil production was elevated by 75% at the Samotlor oil field using 27 wells. Similar improvements in the rate of oil recovery by 20–75% have been shown in several laboratory and field studies [14], [9], [10], [11]. Developments have been witnessed as well in the dehydration of water in oil emulsions, with separation efficiencies between 20 and 95% [15], [16], [17], [18], [19], [20], [21], [22], [23], [24], [25], [26], [27], [28], [29], [30], [31], [32], [33], [34], [35], [36], [37], [38], [39], [40], [41], [42], [43], [44], [45], [46], [47], [48], [49], [50], [51], [52], [53]. Due to these significant developments and numerous research findings in US assisted oil technologies which has seen sustained increment in the past decade, there is a need for a detailed review which highlights the progress and challenges as well as prospects (Fig. 4). This review of recent findings is crucial to further research developments, policy decision making and strategic investments. To our knowledge, there is no detailed review of the potentiality of US assisted technologies such as US enhanced green demulsification, high intensity focused ultrasound (HiFU) and modeling efforts in EOR and oil emulsions dewatering. Moreover, there are some special conditions which have improved dehydration efficiencies and it is essential to highlight these progresses. For instance, Ronchi et al [39] observed that the usage of Raschig rings in the acoustic chamber enhanced the separation of oil from water. Moreover, metallic rings such as copper and steel were described as having better demulsification performance as compared to organic rings such as polyvinyl chloride and poly propylene. In their study, Sadatshojaie et al [30] examined the dehydration of three medium crude oil (Crude oil 020, 030 and 040) of different salt concentrations using a static pipe with a volume of 100 cm^3^. They observed that the more the salt content in crude oil, the better the separation of the water from the oil. At high temperature, Yi et al [38] showed that the equilibrium demulsification efficiency with sonochemistry increased with rising temperature. These findings are crucial to further research progress in US assisted technologies.

Hence, in this review, the developments in the utilization of US either as standalone or integrated with other technologies in EOR and dehydration of water in oil emulsions were analyzed. The studies include the optimization of fluid and US properties in EOR and demulsification. Reports on the treatment of formation damage resulting from inorganic salts, organic scales, drilling fluid plugs, condensate, paraffin wax and colloidal particle with US-assisted EOR were also highlighted. Moreover, the mechanisms were examined in order to gain insightful understanding and to aid research investigations in these areas. Technologies such as US assisted green demulsification, high intensity focused ultrasound, and potential pathways in field studies were assessed for their feasibilities. It is essential to evaluate these technologies due to the significant accrued benefits in them. The usage of green demulsifiers such as deep eutectic solvents, ionic liquids and bio-demulsifiers has promising future outlook and US could enhance their technical advancement. HiFU has been applied successfully in clinical research and developments in this area can potentiality improve demulsification and interfacial studies (fluid–fluid and solid–fluid interactions). As regards field studies, there is need to increase actual well investigations because present reports have few on-site measurements with most studies being in laboratory scale. Furthermore, there is need for more detailed modeling of these technologies as it would assist in conserving resources, saving research time, and fast-tracking oil production. Additional evaluative studies of conditions such as the usage of Raschig rings, crude oil salinity and high temperature which have improved demulsification of crude oil emulsions should be pursued.

## 2 Theoretical background

**Fig. 5:**
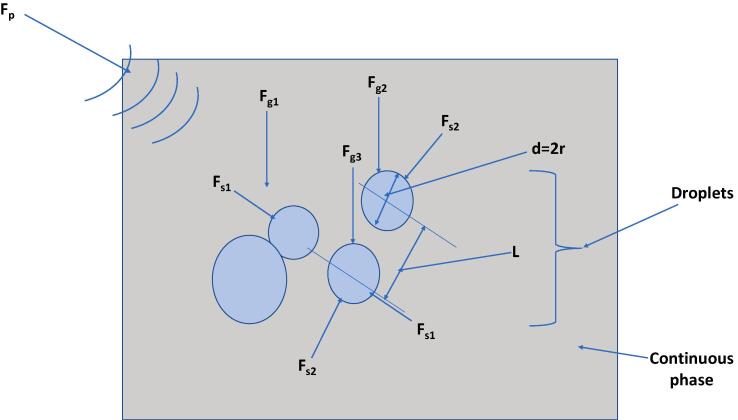
Forces involved in the coalescence of water droplets in crude oil continuous phase.

Ultrasonic waves applications in crude oil treatment and extraction are influenced by a combination of primary acoustic radiation (F_p_), secondary (F_s_) and gravitational (F_g_) forces (Fig. 5). The primary acoustic force comes from the transducers. It allows for the positive contrast droplets of water to move to the nodal points and thus drive their coalescence. Likewise, the antinode receives coalesce of negative contrast droplets. The secondary forces occur as the droplets move closer. Hence, it further enhances the aggregation of the droplets. The gravitational force then allows the large aggregates to settle at the bottom of the chamber.

The primary acoustic radiation force is described based on the equation (1) [39]:

(1) $$F_{p}=-\left(\frac{\pi V}{2\lambda \rho_{o}{c_{o}}^{2}}\right)\phi {p_{o}}^{2}sin\left(2kz\right)$$

(2) $$\phi =\frac{5\rho_{d}-2\rho_{c}}{2\rho_{d}+\rho_{c}}-\frac{\rho_{o}{c_{o}}^{2}}{\rho_{c}{c_{c}}^{2}}$$

Where p_o_ is the pressure amplitude, c_d_ is the droplet sound velocity, c_o_ is the continuous phase sound velocity, ρ_d_ is the droplet density, ρ_c_ is the continuous phase density, Φ is the acoustic contrast factor, k is the wavenumber, z is the droplet position and λ is the acoustic wavelength.

The secondary force, which depends on distance between close droplets, point velocity and pressure values is represented as [39]:

(3) $$F_{s}=4\pi {r_{1}}^{3}{r_{2}}^{3}\left[\frac{{\left(\rho_{d}-\rho_{c}\right)}^{2}\left(3\cos^{2}\theta -1\right)}{6\rho_{c}l^{4}}v^{2}\left(z\right)-\frac{\omega^{2}\rho_{c}{\left(\beta_{d}-\beta_{c}\right)}^{2}}{9l^{2}}p^{2}\left(z\right)\right]$$

Where r_1_ and r_2_ are radii of two nearby droplets, p(z) is the pressure at a specific droplet position, l is the central distance between two nearby droplets, v(z) is the velocity at a specific droplet position, θ is the angle between the droplet centerline and direction of incident sound waves, β_c_ is the compressibility of the continuous phase and β_d_ is the compressibility of the droplet.

The gravitational force is described as in equation (4) [54]:

(4) $$F_{g}=\left(\rho_{d/a}-\rho_{c}\right)Vg$$

Where ρ_d/a_ is the droplet or aggregate’s density, V is the droplet or aggregate’s volume and g is the gravitational acceleration.

Under EOR conditions, the Helmholtz equation (5) describes the propagation of the US [55]:

(5) $$v\left(-\frac{1}{\rho_{p}}\nabla p\right)-\frac{\omega^{2}p}{\rho_{p}c^{2}}=0$$

Where p is the acoustic pressure and ρ_p_ is the phase density.

The longitudinal velocity, which is the relevant velocity under EOR, is represented in equation (6) below [11]:

(6) $$C_{L}=\sqrt{\frac{K+\frac{4G}{3}}{\rho_{p}}}$$

Where G is the shear modulus and K is the bulk modulus.

## 3 Recent progress in the utilization of US in oil technologies

### 3.1 US in demulsification of Water-in-Oil emulsions

The formation of emulsions during the production of crude oil is inevitable. Crude oil production reservoirs usually have significant amount of formation water which could reach 90% and are produced alongside the oil as a mixture [12]. Moreover, water is used in drilling and extraction of oil and the utilization of water becomes more significant with flooding during enhanced oil recovery (EOR) and with increment in the age of the reservoir. The presence of these two immiscible liquids, turbulence generated by flow through constricted pores in the reservoir, chokes at well head, and surface facilities, and amphiphilic surface-active agents in the form of asphaltene, resins, waxes, mineral scales, corrosion products, clay and EOR surfactant such as polymers and alkali leads to the production of very stable oil emulsions. Stable emulsions occur in the form of water-in-oil (W/O), oil-in-water (O/W), water-in-oil-in-water (W/O/W), oil-in-water-in-oil (O/W/O). However, it is mainly encountered as water-in-oil emulsions [13], [14], [15]. These emulsions often cause challenges through extraction, transportation, storage and refining. They cause pressure drop and subsequent increased pumping costs due to their high viscosity [16]. In addition, emulsions could lead to clogging, corrosion and erosion in pipelines during transportation and the poisoning of catalysts in the refining process [16], [17], [18], [19], [20], [21], [22]. Hence, there are many studies that have been reported on the demulsification of crude oil [23], [24], [25], [26], [27], [28], [29], [30], [31], [32], [33], [34], [35], [36], [37], [38], [39], [40], [41], [42], [43], [44], [45], [46], [47].

The approaches that have been pursued in the demulsification process includes biological [23], [24], [25], chemical [26], [27], [28], [29], [30], [31] and physical [32], [33], [34], [35], [36], [37], [38], [39], [40], [41], [42], [43], [44], [45], [46], [47] separation processes. Although biological separation through bacteria or synthetic agents provides better environmental benefits when compared to chemical demulsifiers, the separation process takes significant amount of time that could disrupt oil production. Physical separation such as natural sedimentation through gravity is also faced with extended time of demulsification of the oil. Other physical dewatering processes includes electrostatic, membrane, thermal and ultrasound. The different physical demulsification technologies have environmental benefits over chemical dehydration. However, they encounter various technical challenges. Electrostatic demulsification encounters challenges with the treatment of emulsions with varying properties such as crude oil emulsions with high initial water content [32]. Heating and constant increment of the density of crude oil in thermal demulsification could lead to elimination of crude oil’s light ends and negative impacts on the gravitational settlement [33]. Membranes are often affected by fouling which causes reduced flux, lower lifespan, costly maintenance and subsequent damage of the membrane [34]. Ultrasonic assisted demulsification of oil emulsions could potentially resolve many challenges faced by the other technologies either when used as an integrated addition or as standalone [35], [36], [37], [38], [39], [40], [41], [42], [43], [44], [45], [46], [47], [48], [49], [50], [51], [52].

**Fig. 6:**
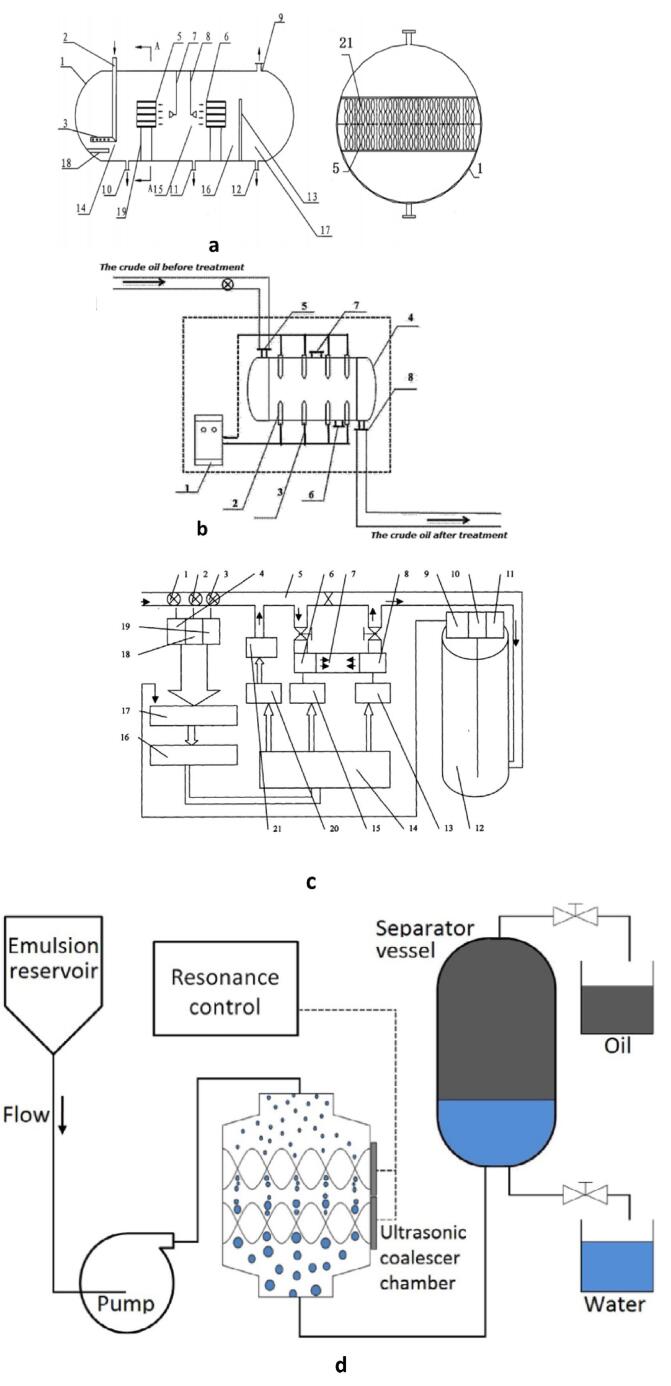
Configuration of different online ultrasonic demulsification devices: **a** US assisted dehydration device for water in oil emulsions separation with secondary re-emulsification suppression capability [37]**b** Power stabilized, time monitored US System for effective and continuous water in oil emulsion dehydration [37]**c** Automatic US supported demulsification device [50]**d** Water in oil emulsion separation aided by an ultrasonic coalescer [45].

Ultrasonic demulsification has advantages of producing high dehydration efficiency which could be>93%, possessing simple and low-cost device and having less hazardous impacts on the environment [35]. The process of ultrasonic dehydration involves the generation of US of varying properties through transducers. The US is then propagated into acoustic chambers where they enhance demulsification through mechanical oscillation, stable cavitation, droplets aggregation and banding. Although there are several experiments that have been conducted under static conditions [36], [37], [38], [39], [40], [41], the dynamic online set-ups allow for undisrupted oil production and could be applied in tanks and pipelines (Fig. 6). Ding and Zhang [42] designed one of such dynamic dehydration device in order to produce stable US power that are devoid of attenuation through a time controlled US cabinet. The device consists of 6 piezoelectric transducers which generates vertical US in a separation tank. The design by Wang and Li [43] has comparable features to that of Ding and Zhang [42] as it has two distinct chambers with individual transducers which produce horizontal US. However, the flow configuration is different as the separation was not conducted in a tank. Rui [44] designed an US assisted demulsification of emulsions in order to resolve the problem of limited area and treatment time of online dewatering processes. The demulsification is performed in two stages with two distinct acoustic chambers placed in a flow tank. Each chamber has a single ultrasonic transducer which produces horizontal US. The demulsification device described by Atehortua et al [45] consists of gravity separator vessel with drainage of the aggregated water, 1 MHz ultrasonic chamber of volume of 264 cm^3^, oil discharge, a hydraulic pump and emulsion reservoir tank. The set-up consists of two piezoelectric ultrasonic transducers with 40 W each at the right-hand side of the chamber. The transducers generate horizontal US which enhances demulsification via gravitational sedimentation of coalescence water droplets.

In the past 5 years, different studies have investigated the ultrasound assisted demulsification of crude oil emulsions in an attempt to optimize the process (Table 1). In one of such studies, Luo et al [53] studied the effect of different acoustic and fluid parameters on the ultrasonic dehydration of water droplets in Silicon oil in order to maximize the separation efficiency. They evaluated the impacts of US energy densities (0–130 J mL^−1^), irradiation time (0–25 min), frequency (25.8–126.4 kHz) as well as amount of water cut in oil (2.5–20%), interfacial tension (4.79–10.35 mN m^−1^) and viscosity (52.19–282.17 mPa s) on the separation efficiency of the emulsion. The experiments were conducted in an acoustic chamber with a volume of 288 cm^3^ and with four piezoelectric transducers with diameter of 35–55 mm and power of 60 W. They attributed the demulsification at low frequencies (25.8 and 39.4 kHz) with mechanical oscillation as the driving mechanism for separation. However, droplet aggregation and banding was the main cause of dehydration at high frequencies (90.0 and 126.4 kHz). Furthermore, they suggested that high frequency US was effective for emulsions with low dispersed water content and small droplet size but low frequency US are suitable for emulsion with high viscosity and interfacial strength. In another study, Luo et al [36] assessed the performance of US demulsification with gravitational sedimentation. They found that US performed better than gravitational sedimentation under the water content (5–25%), interfacial conditions (5–11 mN m^−1^) and irradiation time (0–30 mins) conditions studied. Moreover, US performed better than gravitational sedimentation at viscosities lower than 200 mPa s, after which the difference in demulsification was reduced until they become the same at ∼ 535 mPa s. Overall, separation was better at lower oil viscosity and higher interfacial tension under ultrasonic separation. Excessive acoustic intensity is undesirable in both low (I > 2.03 W cm^−2^ at 18.96 kHz) and high (I > 2.5 W cm^−2^ at 126.34 kHz) frequency US. Sadatshojaie et al [41] studied the dehydration of three medium crude oil (Crude oil 020, 030 and 040) of different salt concentrations using a static pipe with a volume of 100 cm^3^. The set-up consists of two horn type piezoelectric transducers placed at both ends of the pipe to send US at 20 kHz into the separation domain. Sensitivity of parameters such as US intensity (0.25–1 W cm^−3^), water cut in emulsion (10–25%) and irradiation time (0–5 min) on the demulsification efficiencies was observed. The results showed that increased initial water content and irradiation time lead to enhanced dehydration of the emulsions. However, the dehydration was less effectiveness at water cuts of 20 and 25%. In addition, the separation performance of the emulsion reduced at irradiation time>2 min from ∼ 85% to ∼ 83% at 5 min. Moreover, the more the salt content in crude oil, the better the separation of the water from the oil. Atehortua et al [45] studied the dewatering of oil emulsions using a 1 MHz ultrasonic coalescence chamber under flow conditions. The parameters evaluated include flow rate (50, 100 cm^3^ min^−1^), extent of US supplementation, concentration of chemical demulsifier (25, 50 ppm), flow inlet temperature (60, 70 °C) and water content of emulsions (30, 50%). They showed that demulsification was enhanced with the utilization of US, lower flow rate and more initial water cut. Temperature did not have any significant effect on the dehydration process. Xu et al [37] investigated the effect of US dehydration of SAGD watery crude oil with demulsifier of 250 ppm. Similar to the findings of Aterhortua et al [45], they demonstrated that the utilization of US in demulsification can lower the demulsifier concentration in chemical demulsification and settling time as compared to natural sedimentation by ∼ 50%. High initial water content and power lower than a critical value of 100 W produced enhanced dewatering of the emulsions, reducing the water cut to ∼ 1.5% after utilizing the US. However, better demulsification was observed at low temperature as against the findings of Atehortua et al [45] who reported little effect of temperature. The differences could be attributed to the varying properties of the oil, the relatively lower frequency and the static flow conditions in the study of Xu et al [37].

**Table 1:** Summary of findings on the demulsification of oil emulsions with US.

No	Probe Description/Assessment	Experimental Parameters	Key Findings	Reference
1	- 4 various Piezoelectric Transducers at different frequencies used - -Frequencies: 25.8, 39.4, 90.0, 126.4 kHz - -Intensity: 60 W - -Transducer diameters: 55, 46, 42, 35 mm	- Silicone oils (Dow Corning Co) - Emulsion properties: viscosities 52.19, 106.00, 169.50, 282.17 mPa s at 20 °C; water content in oil: 2.5, 5, 10, 20% used - Emulsion volumes: 201.6, 176.4, 172.8, 180.0 mL	- Mechanical oscillation is the main cause of emulsion separation for low frequency US (25.8 and 39.4 kHz), whilst droplet aggregation and banding is the main route for high frequency US (90.0 and 126.4 kHz) - High frequency US effective for emulsions with low dispersed phase content, less energy density compared to low frequency US and small droplet size but low frequency US are suitable for emulsion with high viscosity and interfacial strength	Luo et al, 2020 [53]
2	- Horn type piezoelectric transducer - Frequency: 20 kHz - Power: 25, 50, 75, 100 W	- Crude oil 020, 030, 040 with viscosities 19, 20, 21 mm^2^ s^−1^, respectively and initial water content (10, 15, 20, 25%) were used - Effect of ultrasonic field intensity (0.25, 0.5, 0.75, 1 W cm^−3^) and irradiation time (0–5 min) on the dewatering process was studied	- Although higher initial water content and irradiation time provided better de-emulsification, there is less effectiveness at 20 and 25%; and separation reduced at irradiation time>5 min - The more the salt content in crude oil, the better the separation of the water from the oil	Sadatshojaie et al, 2021 [41]
3	- -Piezoelectric Transducers used - -Frequencies: 18.96, 126.34 kHz - -Intensity: 60 W	- Silicone oils (Dow Corning Co) with viscosities 52.19, 106.00, 169.50, 282.17, 378.52, 530.20 mPa s and different water contents in oil (2.5, 5, 10, 20, 25%) were evaluated - Total oil–water emulsion volume = 100 mL - Interfacial tension: 5.12–11.18 mN m^−1^	- Excessive acoustic intensity is undesirable in both low (I > 2.03 W m^−2^) and high (I > 2.5 W m^−2^) frequency US - US performed better than gravitational sedimentation under the water content (5–25%), interfacial conditions (5–11 mN m^−1^) and irradiation time (0–30 mins) conditions studied. - US performed better than gravitational sedimentation at viscosities lower than 200 mPa s, after which the difference in demulsification was reduced until they become the same at ∼ 535 mPa s	Luo et al, 2019 [36]
4	- Two piezoelectric transducers - Individual power: 40 W - Ultrasonic coaleser chamber of 1 MHz was achieved with the transducers at the side of the chamber. The chamber has a volume of 264 cm^3^	- Oil composed of 70% of oil 29 API and 30% oil 13 API (Vol) with viscosities of 24, 15 cP at 60, 70 °C, respectively - Water content of 30, 50% - Amount of demulsifier: 25, 50, 100 ppm	- Ultrasonic coalescence showed potential to lower separation time as compared to gravitational segregation - Under integrated US and chemical demulsifier conditions, the US reduced the consumption of chemical demulsifiers to 25 ppm as compared to 50 ppm in standalone chemical demulsification. Hence, it lowers production costs - The demulsification efficiency was similar at 60 and 70 °C	Atehortua et al, 2019 [45]
5	Piezoelectric transducer at 100 W	- SAGD watery crude oil used - Type SD demulsifier at 250 ppm - Effect of US irradiation time and temperature on demulsification, and comparative evaluation US assisted technologies were assessed - Temperature of 40, 50, 60 and 70 °C used	- Usage of US in demulsification can lower the demulsifier concentration in chemical demulsification and settling time as compared to natural sedimentation - Better demulsification was observed at low temperature, high water content and power lower than a critical value of 100 W	Xu et al, 2019 [37]
6	- Three different non contacting meter transducers used - Power: 50, 100 and 150 W - Frequency: 40, 30 and 20 kHz - Chamber volume: 100 mL	- Watery crude oils emulsion from Daqing Oilfield with water content of 25.23%, density of 0.9247 g cm^−3^, viscosity of 145000 mPa s, and salt content of 95367 mg L^-1^ was used	- -Dehydration via sonochemistry (∼45%) performed better than standalone ultrasound (20%) and chemical (34%) methods - -The equilibrium demulsification efficiency with sonochemistry increased with rising temperature and power	Yi et al., 2017 [38]
7	- Ultrasonic water bath at adjustable frequency 25, 45 and 135 kHz used - Power: 100, 200 W	- Heavy crude oil with water content of 0.41%, density of 0.9574 g cm^−3^ at 20 °C, kinematic viscosity of 1195 mm s^−2^ at 60 °C and salt content of 0.41% utilized for the emulsion synthesis - Effect of Raschig rings in the acoustic chamber, ultrasound frequency and temperature on the separation of the oil–water emulsion was observed	- -Usage of Raschig rings in the acoustic chamber enhanced the separation of oil from water. - -Metallic rings such as copper and steel was described as having better demulsification performance as compared to organic rings such as polyvinyl chloride and poly propylene - -Demulsification was improved by increasing the ultrasonic waves irradiation time and frequency. However, the effect of time stabilized after 15 min for 35 and 45 kHz - -The optimum irradiation time at 135 kHz was 20 min	Ronchi et al, 2020 [39]
8	- Vessel dimensions: - Length 25 cm - Diameter 4.8 cm - Two transducers at each end of vessel with frequency of 20 kHz - Power: 25, 50, 75, 100 W	- Three crude oils Cheshmeh Khosh, Gachsaran 1 and Gachsaran 2 with kinematic viscosities between 19.4 and 21.4 mm^2^ s^−1^ at 25 °C were used - -Effect of water cut of emulsion (10–25%), ultrasonic irradiation time (0–5 min) and intensity (0–1 W cm^−3^) on demulsification effectiveness investigated	- -Increment in ultrasonic irradiation time provided better dehydration than increment in the intensity - -For the crude oils studied, the authors suggested that the utilization of chemical demulsifiers could be reduced by 50% at suitable US intensity and time	Khajehesamedini et al, 2018 [40]
9	- Ultrasonic bath - Power: 100, 160 W - Frequency: 35 kHz	- Crude oil with API density of 19.0 and viscosity of 122.9 mPa.s at 45 °C used - Effect of parameters such as water content (12, 35, 50%), time (5–60 min) and temperature (25, 45, 60 °C) on dewatering efficiency evaluated	- -Dewatering efficiency attains 65% at droplet size of 10 µm at water cut of 50% - -Good separation efficiency (51.7%) was achieved in 15 min at US power of 160 W and US temperature of 45 °C	Antes et al, 2015 [51]
10	- Ultrasonic bath - US Intensity Amplitude: 20, 60, 100% - Frequency: 35 kHz	Emulsion contains crude oil with viscosity of 279 cP and API density of 19.7; water droplet of median size (5 µm) and NaCl concentration of 250 g L^-1^	- Through 3D transitioning hydrophones, the mapping of the US bath was achieved across its width (240 mm), depth (150 mm) and length (300 mm) - The acoustic intensities across the US bath ranges from 0.1 to 0.6 W cm^−2^	Pedrotti et al, 2018 [17]
11	- Ultrasonic bath - Power: 100, 200 W - Frequency: 25, 35, 45, 130, 582, 862, 1146 kHz	Emulsion with crude oil of API density of 19 and water cut of 12, 35, 50%Water droplets of median sizes (5, 10 and 25 µm) were used	- About 65% separation efficiency after 15 min at 10 µm droplet diameter, 50% water content and f = 45 kHz achieved - No apparent demulsification at frequencies greater than 45 kHz	Antes et al, 2017 [21]

#### 3.1.1 Mechanism of ultrasonic demulsification

**Fig. 7:**
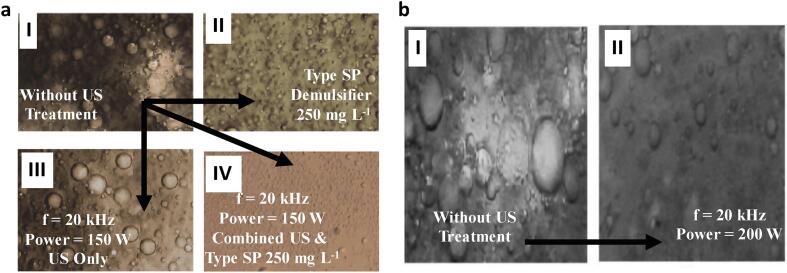
The mechanism of ultrasonic demulsification: **a** Effect of US with and without chemical demulsifiers [38]**b** Effect of US on super heavy crude oil emulsion [50].

The mechanism of demulsification have been described to occur through mechanical vibration, stable cavitation, droplets aggregation and banding (Fig. 7). Luo et al [53] described droplet migration and aggregation at pressure node which is followed by droplet banding at high US frequencies. This phenomenon of aggregation and banding is disrupted by acoustic streaming associated with increased energy densities. At lower frequencies, water droplet was shown to approach, collide and coalesces in the presence of bubble vibration caused by stable cavitation under low acoustic intensities. However, as the intensities increase beyond a critical value, the cavitation bubbles explode and lead to secondary emulsification. Hence, the authors stated that intense cavitation and acoustic streaming should be eliminated so as to attain optimum dewatering process. Similar effect was highlighted by Luo et [36] in another study where oil droplets aggregate into clusters. Sadatshojaie et al [41] reported similar findings of water droplets coalescing in continuous oil phase.

In their study, Yi et al [38] highlighted that the initial crude oil several droplets with varying diameters behaved differently under various treatment technologies. With the application of standalone chemical demulsification, the amount and size of the water droplet reduced as compared to the untreated emulsions. Conversely, the water droplets under ultrasonic waves were larger than chemical demulsification. The authors suggested that this imply the better separation effect of demulsifiers. Under a combination of US and demulsifier, the number of water droplets was significantly reduced and the droplets have lower diameters compared to stand alone chemical and US demulsification. Similar behavior of water droplets could be found in the work of Yang et al [18] and Ye at el [20]. Ye et al [20] suggested that the reduction in the number of droplet particles which is less than 3 µm lowered the possibility of droplet collision and thus slowed down demulsification and elongated time. In agreement with other studies, Wang et al [50] noticed that both the diameter and amount of water droplets reduced after the application of US to water-in-oil emulsions. However, Xie et al [48] studied the impact of US in the demulsification of water in oil emulsions and observed a different phenomenon. They showed that the water droplets increased in diameter but decreased in number under US condition.

**Fig. 8:**
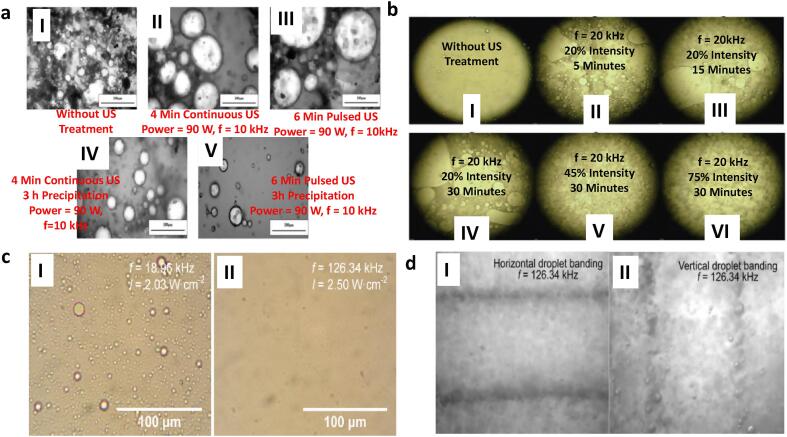
Effect of ultrasonic transducer parameter on the mechanism of demulsification: **a** Pulsed vs Continuous US [48]**b** Creaming effect under different US intensities and time [19]**c** Low vs High Frequency [36]**d** Horizontal vs Vertical Banding [36].

Furthermore, the effect of different parameters on the mechanism of demulsification was studied by several groups (Fig. 8). For example, Xie et al [48] further showed that pulsed US produced less droplets after precipitation which were more obvious as compared to continuous US. Nasiri et al [19] described the morphological changes in emulsions under different time (5–30 min) and intensities (20, 45 and 75%). They showed that the preliminary emulsions which were stable and consists of small droplets transformed into aggregates as the time progressed. The coalescence rate increased until 30 min, at which optimum instability and separation was achieved. As regards the intensity, the separation process was dominated by droplet creaming whose rate increased to 41.67, 52.08 and 72.3% at 20, 45 and 75% intensities, respectively. Although separation efficiency at low frequency (18.96 kHz) is a little more than at high frequency (126.34 kHz), water droplet aggregates at 18.96 kHz contains significant amount of small oil droplets (Luo et al, [36]). The water aggregates at 126.34 kHz showed no apparent oil droplets. The presence of oil droplets in separated water was attributed to the unstable interface between the water and oil, and the formation of tiny oil droplets due to cavitation bubbles collapse. The effect of US direction (horizontal and vertical) on the droplet aggregation, banding and subsequent dehydration was studied (Luo et al, [36]). Whilst the primary acoustic force in horizontal US propagation was perpendicular to gravity, the vertical US was opposite to the gravitational effects. The orientation of the horizontal US allows for droplet aggregation and banding that occurs simultaneously with sedimentation. However, vertical US have delayed sedimentation and less separation because their primary acoustic forces are countercurrent to gravitational forces. In addition, horizontal US have more narrow bandings that could improve droplet coalescence and the droplet depositions does not impede with the US propagation as compared to vertical US.

In their study, Pangu and Feke [125] examined the relative trajectory and mechanism of coalescence of two oil droplets in an aqueous continuous phase under the application of US waves. In addition, the time transformation of the adjacent fluid due to the influence of the monitored droplet was observed under different conditions of the acoustic field, droplet size and location, as well as the physical properties of the dispersed and continuous phases. The droplets’ motion and subsequent coalescence were influenced by body forces (primary acoustic forces and net gravitational-buoyancy force) and inter-droplet forces (secondary acoustic forces, van der Waals forces and hydrodynamic association). Taking into account the various forces that act on the droplets, the relative motion was predicted with the Batchelor equation [126].

(7) $${\overset{-}{V}}_{12}=V_{12}^{0}\left(-Lcos\theta {\hat{e}}_{r}+Msin\theta {\hat{e}}_{\theta }\right)-\omega_{12}^{0}G\left(\frac{dV_{\mathrm{vdW}}}{\mathrm{dr}}+F_{2,ac}\right){\hat{e}}_{r}$$

Where ${\overset{-}{V}}_{12}$ is the relative droplet velocity, $V_{12}^{0}$ is the relative velocity contribution from body forces, L and G are the axisymmetry relative mobility function, M is the asymmetric relative mobility function, θ is the relative angle to the vertical axis, $\omega_{12}^{0}$ is the relative hydrodynamic mobility, $V_{\mathrm{vdW}}$ is the van der Waals potential, r is the distance between the droplets’ centers, $F_{2,ac}$ is the secondary acoustic force, ${\hat{e}}_{\theta }$ and ${\hat{e}}_{r}$ are the unit vectors along the tangential and radial directions.

The transition of the droplets to equilibrium positions and subsequent collision and coalescence progressed in two stages. Initially, the droplets moved quickly towards the equilibrium location close to the acoustic pressure antinode. This swift movement of the droplets was associated with the influence of the primary acoustic and net gravitational forces. Subsequently, at the equilibrium position, the droplets slowed down, collided and coalesced through the van der Waals and secondary acoustic forces. This behavior of droplets starting with a fast approach, and then slowing down at the collection plane before collision and coalescence has been reported by several other groups as well [52], [54], [127]. In a different study, Pangu and Feke [54] discussed that the droplets within a width of λ/2 from the collection plane would transition to the plane which is nearby to the pressure antinode swiftly through the body forces. On reaching the equilibrium plane, the droplets slowed down and the relative angle to the vertical axis θ becomes $\frac{\pi }{2}$ which eliminates the impact of the body forces. Although the coalescence of the droplets at the equilibrium position was generally due to the inter-droplet forces, the main driver of the aggregation was the secondary acoustic force. At small droplet diameter of 10 µm, the authors demonstrated that more volume rate was cleared at low frequency (0.525 MHz) as compared to higher frequency (1.69 MHz) by the secondary acoustic forces. However, with larger droplets (20 µm) increased frequency of 1.69 MHz was favorable for coalescence. In the description of the mechanism of droplet separation from the continuous phase, Luo et al [127] explained further on the collection of droplets at the equilibrium positions. They highlighted that the aggregation of the droplets at the band region (width = λ/8) occur at the pressure antinode if the acoustophoretic coefficient (K_s_) is greater than zero. In the case of an acoustophoretic coefficient less than zero, the aggregation of the droplets happens at the collection plane close to the pressure node. Furthermore, the collection at the band region occurs if the droplets are composed of the same fluid type. For different fluid droplet types, the separation mechanism of the droplets would proceed based on differences in the acoustophoretic coefficients (K_s_ less than 0 or K_s_ > 0) or density if the K_s_ values of the droplets are of the same order. The coalescence and separation of droplets could be inhibited by acoustic streaming and cavitation. Acoustic cavitation is the process of initiation, growth, and the subsequent collapse of bubbles by shock waves. This phenomenon is attributed to the unstable nature of the boundaries of water–oil interface with the application of US waves. In the case of water, the cavitation threshold can be reached when the distance of the van der Waal (4 × 10^-10^ m) is lower than the water molecules distance. Acoustic streaming was also the subject of other studies among them [127], [128], [129], [130], [131]. In particular, streaming event has been described to cause the disintegration of droplets and the destruction of their collection band [127].

### 3.2 US in EOR

**Fig. 9:**
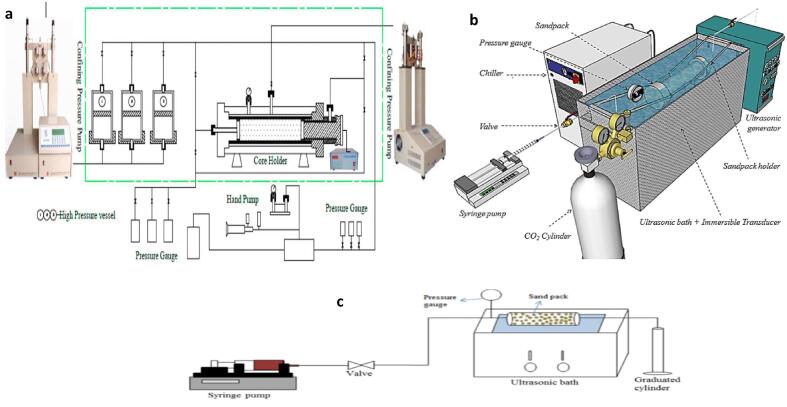
Experimental set-ups for enhanced oil recovery with US and flooding: **a** US Supported Plug Elimination Device (Power = 1000 W, f = 18, 20, 25, 30, 40, 50 kHz) [71]**b** Micro-model apparatus for carbon dioxide flooding of core samples with and without US application towards improved oil recovery assessment (Power = 500 W, f = 40 kHz) [69]**c** Water Flooding Device with US Irradiation Capacity (Frequency = 37 kHz, Power = 150 W) [61].

Tertiary enhanced oil recovery technologies are essential because the reservoir pressure drops and significant formation damage develops over extended period of time. This results in tremendous limitation in the amount of oil that could be recovered from the reservoir after primary and secondary extraction. Some studies have estimated that 60–67% of crude oil remains after secondary oil recovery [54], [55], [56], [57]. Hence, research studies on further recovery of the residual oil is essential. The utilization of US is one of the various technologies that have demonstrated potential capability to recover these residual oils (Fig. 9) [58], [59], [60], [61], [62], [63], [64], [65], [66], [67], [68], [69], [70]. Agi et al [58] evaluated the effect of acoustic parameters such as US type (intermittent or continuous), US power (150 and 500 W) and distance to the micromodel (15 and 30 cm) on the enhancement of the recovery of kerosene and paraffin. The US frequency of 40 kHz was used. The square micromodel (60 mm by 60 mm) used has permeability of 1.94D, porosity of 40%, throat diameter of 0.1 mm and pore volume of 37.69 mm^3^. They detected that US improved the recovery of kerosene and paraffin by 4% and 50%, respectively. Intermittent US performed better than continuous US in the recovery of the oils. For kerosene, intermittent and continuous US produced 42% and 32% improvement in the recoveries, respectively. With the paraffin, recoveries of 70% and 55% were achieved for intermittent and continuous US application, respectively. Moreover, high US power (500 W) and closer transducer distance to micromodel (15 cm) generally produced better performance. For instance, heavy oil at 30 cm showed 68% recovery at 500 W and 60% recovery at 150 W. However, at 15 cm the recovery increased to 69% at 500 W and 65% at 150 W. Dehshebi et al [60] found similar behavior for US applied in the enhanced oil recovery of two crude oils with viscosities of 9.57 and 88.22 cP in a micromodel. The recovery of the oils was improved by up to 40%. Lighter oils witnessed better recoveries of about 1.2–3 times that of heavy oils. In a different study, Agi et al [59] investigated the effect of US in water and surfactant flooding. Ultrasonic bath of dimensions 21 cm by 50 cm by 30 cm with frequency of 40 kHz and US power intensities of 150, 300 and 500 W cm^−2^ was utilized. Sandpack was used as the porous media and was placed at the center of the US bath to allow for optimum irradiation. Centrifuge pump was used to channel fluid flow into the sandpack while the vacuum pump evacuates the fluid before saturation. US was shown to improve the paraffin recovery for water and surfactant flooding by 11% and 12%, respectively. They attributed the enhancement from the US to the thermal effect which resulted in viscosity reduction, formation of micelles and intermolecular impact of the US. The oil recovery exhibited increment as the power intensity was raised from 150 W cm^−2^ (61.1%) to 500 W cm^−2^ (67.4%). The effect of US in enhancing paraffin oil recovery in a tertiary carbon dioxide flooding in a porous media was investigated by Hamidi et al [69]. Cylindrical sandpack with a length of 20 cm, radius of 2.5 cm, porosity of 28%, pore volume of 109.95 cm^3^ and containing sand radius of between 30 and 40 µm was used to represent the porous media. Immersible transducer with output power of 500 W, frequency of 40 kHz and calorimetric efficiency of 35.4% produced US in a bath of dimensions 21 cm by 50 cm by 30 cm. The effect of carbon dioxide injection rate (2, 3.5, 5 and 10 cm^3^ min^−1^) and controlled vs uncontrolled temperature conditions was observed. Better recoveries of the paraffin oil were obtained as the CO_2_ injection rate increased. Moreover, uncontrolled temperature condition performed better than controlled temperature condition. Consequently, maximum paraffin recovery of 40.9% was attained with uncontrolled temperature condition at CO_2_ injection rate of 10 cm^3^ min^−1^.

**Fig. 10:**
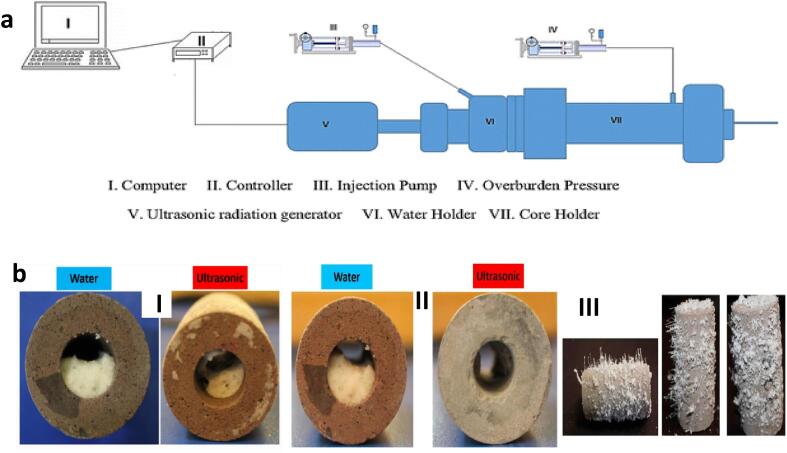
Removal of inorganic scales in near wellbore region with US: **a** Ultrasonic flooding set-up (f = 22 kHz, Power = 1000 W) [74]**b I** Unplugging of sodium chloride deposit within a core sample through the injection of water with and without US treatment [74]**II** Unplugging of potassium chloride deposit within a core sample through the injection of water with and without US treatment [73]**III** Saturated Core Sample with Potassium Chloride Before Treatment [73].

There are several studies that have attempted to improve the permeability and pore structure of porous media which have been damaged, and hence, improve the oil recovery of the reservoir [71], [72], [73], [74], [75], [76], [77]. Some of these studies have focused on the removal of inorganic salts causing formation damage in the near wellbore region (Fig. 10). One of such studies was reported by Khan et al [71] where they investigated the effect of US on the removal of calcium carbonate in the near wellbore region. The inorganic salt elimination was conducted experimentally using the set-up in Fig. 10. In order to optimize the calcium carbonate elimination, three different core samples with distinct pore structures, which were each made of quartz, was utilized. The core samples have a length of 6 cm, radius of 1.25 cm, permeability within 300–1500 µ cm^2^, porosity within 20–24%, density of 1670 kg m^−3^ and pore volume within 5.88–7.06 cm^3^. Moreover, six piezoelectric transducers were used to observe the effect of frequency (18, 20, 25, 30, 40, 50 kHz), power (60, 200, 1000 W) and irradiation time (60, 80, 100, 120 min). They observed that the optimum US power and frequency are 1000 W and 20 kHz, respectively. Under this condition, the peak recoveries of the core permeability were 35.7%, 36.3% and 34.1% at starting core permeabilities of 1500, 800 and 300 µ cm^2^, respectively. In addition, peak carbonate plug elimination from the near well bore was achieved at 1 h 40 mins, reaching core permeability recovery efficiencies of 38.1%, 37.5% and 35.9% with initial core permeabilities of 1500, 800 and 300 µ cm^2^, respectively. Zhang et al [72] conducted experiments in an attempt to eliminate the calcium carbonate in core samples through the application of US and chemical treatment. Three cores with different pore properties and six transducers with different frequencies (18, 22, 25, 30, 40 and 50 kHz) and power (100, 200 and 1000 W) was utilized. The transducers have capacity to withstand a maximum pressure of 35 MPa and temperature of 110 °C. The core sample, which is made of quartz, clay, carbonate and feldspar, have lengths of with 7–8 cm, diameter of 2.5 cm, porosities within 18.9–21.9% and initial core permeabilities between 350 and 1600 µ cm^2^. The core recovery efficiency of the three samples reached 37.98%, 37.92% and 34.23% at 1 h after which it stabilizes between 1 h 20 min – 2 h. The recovery rate of the permeability increased with increasing US frequency and power. The optimum power and frequency are 1000 W and 20–25 kHz at which the peak core permeability recovery rate was 37.98%. In comparison to chemical treatment with hydrochloric acid of 10% concentration, the performance of US was slightly lower in terms of the core permeability recovery efficiency. The authors suggested the increment in the US power in order to achieve better efficiency. However, increasing the US power above 1000 W has been reported to collapse core samples by Taheri Shakib et al [73].

Taheri-Shakib et al [73] studied the displacement of potassium chloride (KCl) scales from near wellbore region through water flooding with and without US. Twenty different core samples with various weight (114.29–199.78 g), diameter (3.747–3.869 cm), length (4.783–7.382 cm), density (2.651–2.834 g cm^−3^), porosity (10.426–27.916%), pore volume (7.697–23.906 cm^3^) and permeability (11.9–2004.7 md) were saturated with crystals of KCl and utilized to replicate conditions in the near wellbore. The core saturation was achieved in a high-pressure cell (27.58 MPa) for 96 h with the concentration of KCl being 220,000 mg L^-1^. The sandwich cylindrical transducer was used at power of 1000 W, 2000 W and 3000 W, and frequency of 22 kHz. However, only the 1000 W was utilized further as higher power resulted in the destruction of the core samples. The integration of US with water flooding significantly enhanced the removal of the KCl crystals from the core, which is more noticeable at lower permeabilities. At initial core permeabilities which were less than 20 md, the recovery efficiency of the water flooding with and without US was 71% and 13%, respectively. Similar observation was detected at other initial core permeabilities though the recovery was less evident. For example, the restoration of the permeabilities were 76% and 44% with water flooding with and without US, respectively. The better performance of core sample scale elimination with US was attributed to the disintegration of KCl through perturbation. This disintegration coupled with high temperature induced by US allows for enhanced brine dissolution in the inhibitory small throat region. Furthermore, combined water flooding and US treatment created wormholes in the KCl crystals which improved the scale withdrawal. Similar behavior of integrated water injection and US for the treatment of NaCl scales was reported by Taheri-Shakib et al [74] in a different study. They showed that the water flooding with US increased the permeability recovery as compared to water injection, with the enhancement reaching 51% in some cases. Increased solubility and scale micro-fractures were detected in the treatment of NaCl saturated core samples as well.

Wang and Huang [75] investigated the rectification of formation damage in core samples which was caused by water sensitivity. They utilized three cores with different pore properties, 10% hydrochloric acid for the chemical treatment and six transducers with different frequencies (18, 22, 25, 30, 40 and 50 kHz) and power (100, 200 and 1000 W). The utilization of chemical, ultrasonic and combined chemical/ultrasonic treatment was assessed. In agreement with the report of Zhang et al [72], increment in the US power and lower frequency increased the core permeabilities reaching optimum values at US power and frequency of 1000 W and 20–25 kHz, respectively. Typical core permeability efficiencies of 24.3%, 23% and 22.1% were obtained in this region. As regards the irradiation time, 100 min was the treatment time to achieve maximum core recovery, reaching 22–25% for the core samples. Whilst the chemical treatment alone was slightly better as compared to standalone US, the integrated chemical and US produced enhanced core permeabilities of 10–20% better than both chemical and US standalone treatments. Khan et al [76] applied similar conditions to those reported by Wang and Huang [75] and observed comparable results. The optimum US power and frequency were 1000 W and 20 kHz, respectively. This produced a peak core recovery of 22.3%, 21.7% and 19.5% with initial permeabilities of 1500, 800 and 300 µ cm^2^, respectively. Optimum treatment time was 100 min with core recovery of 24.2–25.3% for the core samples. Standalone chemical, standalone US and combined chemical and US lead to core permeability recovery efficiencies of 26.1–34.2%, 24.2–25.3% and 43.1–45.8%, respectively for the core samples evaluated. Ghamartale et al [77] studied the effect of US in enhancing the permeabilities and pore structure of five different rock samples. The samples evaluated are Gray dolomite, Oolitic limestone, Indiana limestone, Gray Sister Berea sandstone and Berea sandstone. The samples have diameters between 3.77 and 3.85 cm, lengths between 4.65 and 4.92 cm and porosity of 11.99–21.27%. US with a power of 300 W and frequency of 20 kHz was used. Although the US irradiation enhanced the pore structure for the limestone rock samples, it deteriorated the permeabilities for dolomite and sandstone. For instance, Indiana limestone showed improvement in the core permeabilities by 12.18–25.17% under a confining pressure of 500 psi. But Gray dolomite showed a reduction in permeabilities by up to 67%. The deteriorating effect of US on dolomite was attributed to their heterogeneity, crystallinity and density which prevented the pore network enhancement. The inhibitory role of US in the sandstone was linked to the reduced particle migration due to the clogging of the pore throat. Conversely, limestones have fragile structure which improved micro-fracture, pore network and fine migration.

**Fig. 11:**
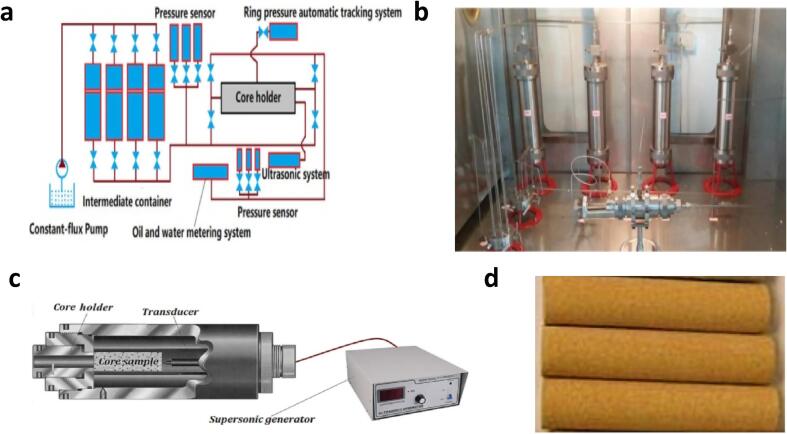
Displacement of organic scales, colloidal particles and condensates from core samples: **a** Simulated Improved Oil Recovery Coupled with US system (Power = 100, 200, 1000 W, Frequency = 18, 22, 25, 30, 40, 50 kHz) [79]**b** Experimental set-up of the displacement device [72]**c** Precipitate Unplugging Device (Power = 100, 200, 1000 W, Frequency = 18, 22, 25, 30, 40, 50 kHz) [79]**d** Artificial Core Sample [79].

There are evaluations of the effect of US on the removal of organic scales, drilling fluid plugs, condensate, paraffin wax and colloidal particle in other studies (Fig. 11). Xu and Bao [78] examined the removal of asphaltene deposit in the near wellbore region using US, chemical and sono-chemical processes. Core samples with initial permeabilities of 300, 800 and 1500 µ cm^2^ was used. In addition, the impact of six piezoelectric transducers with power of 100, 200, 1000 W, frequencies of 18, 20, 25, 30, 40, 50 kHz, and irradiation time of 0–140 min on the improvement of the pore structure was observed. The chemical agent was composed of 10% hydrochloric acid and 5% mud acid. Core displacement system was utilized in applying a ring pressure of 0–50 MPa on the core samples. Lower frequencies (20–25 kHz) were shown to provide the best pore structure enhancement with pore permeability recovery efficiencies of 14.8%, 17.5% and 20.2% achieved for the three core samples studied. The maximum treatment time for the core samples with initial permeabilities of 300, 800 and 1500 µ cm^2^ were 100, 100 and 120 min, respectively. Under these irradiation times, the highest permeability efficiencies were 21.3%, 25.2% and 27.5% for the three core samples. They noted that the improvement in the permeabilities of the core with US decreased from 22.5 to 15.8% under initial permeabilities from 300 to 1500 µ cm^2^. However, the enhancement of the core pore structure increased under chemical (18.5% to 24.8%) and sono-chemical treatment (36.8% to 39.8%) with similar starting pore permeabilities. Generally, sonochemistry (36.8–39.8%) has better pore structure formation as compared to chemical (18.5–24.8%) and US (22.5–15.8%) treatments for the core samples.

Mo et al [79] studied the withdrawal of colloidal precipitates from near-well plug using US with similar set-up utilized by Xu and Bao [78]. Piezoelectric transducers with power of 100, 200, 1000 W, frequencies of 18, 20, 25, 30, 40, 50 kHz, and irradiation time of 0–120 min were used. The core samples have initial permeabilities of 30, 80 and 150 mD. Whilst the improvement of the pore structure increased with US power increment, it degrades at higher frequency. Optimum conditions of US power of 1000 W, irradiation time of 120 min and frequency of 25 kHz was noted. Under these conditions, the peak permeability recovery efficiencies were 26.8%, 28.5% and 32.9% for the three core samples. The removal of the colloidal precipitates from the plug with the application of US can be attributed to acoustic streaming [132]. Karimi et al [82] observe as well that increase in the US power enhanced the removal of condensate blockages. The condensate displacement efficiency increased from 7.85% to 19.21% as the US power was raised from 260 W to 1300 W. Mo et al noted that sono-chemical method provided better pore structure of almost 50% as compared to US and chemical treatment alone. The sono-chemical method produced synergistic effects with the US improving the chemical activity and degradation of the particulates and the chemical process modified the force state of the particulates. Similar observations have been reported by Zhang et al [72] and Xu et al [80]. Yeh and Juarez [81] evaluated the effect of US frequency (25, 40, 55, 70, 85 and 100 kHz) on the diffusion of colloidal particle through porous media using a micromodel. Three types of micromodels with porosities of 0.62, 0.76 and 0.90 was used. Generally, the micromodel has a cylindrical channel (length of 17 mm and width of 2 mm) with several pillars of uniform diameters acting as pore obstruction media. The optimum frequency for the average velocity and diffusion of the colloidal particle occurred at a natural frequency of 40 kHz after which it reduced to a minimum natural frequency at 70 kHz for cores studied with the micromodel. The diffusion of the particles was enhanced by the US, producing a peak diffusion of 0.4 µm^2^ s^−1^ as compared to that without US with 0.073 µm^2^ s^−1^. Zhou and Wang [83] discovered comparable trend to those reported by Mo et al [79] in the removal of paraffin wax from core plugs. Lower frequency (25 kHz) and higher US power (1000 W) was favorable in the displacement of the paraffin wax. The maximum irradiation time was 120, 120 and 140 min at initial core permeabilities of 30 mD, 80 mD and 150 mD, respectively. The core permeability improvements were 23.5%, 27.6% and 31.0% for the three cores. In their study, Wang et al examined the impact of sonochemistry on the withdrawal of paraffin deposit, polymer plug, drilling fluid plug and inorganic scales as compared to US and chemical agents. The chemical agents used includes carbon tetrachloride of 10% concentration for paraffin deposit, mud acid of concentration of 5% for drilling fluid plug, chlorine dioxide of concentration of 5% for polymer plug and hydrochloric acid with concentration of 10% for inorganic scale. They reported that sonochemistry performed better than chemical agents and US by 10–30%.

## 4 Prospects and future trend

Significant developments have been made in the demulsification of medium and heavy crude oil emulsions and improved oil recovery under the application of US. Effect of various US parameters and emulsion properties have been investigated. Generally, enhanced US treatment occurred with less frequency, acoustic intensity lower than critical value, increased irradiation time, pulsed US and lower viscosity. Further research needs to be conducted on unique conditions such as Raschig rings impacts, high temperature and salinity as there are reports that have indicated that they affect US performance. For instance, Ronchi et al [39] observed that the usage of Raschig rings in the acoustic chamber enhanced the separation of oil from water. Moreover, metallic rings such as copper and steel were described as having better demulsification performance as compared to organic rings such as polyvinyl chloride and poly propylene. In their study, Sadatshojaie et al [41] examined the dehydration of three medium crude oil (Crude oil 020, 030 and 040) of different salt concentrations using a static pipe with a volume of 100 cm^3^. They observed that the more the salt content in crude oil, the better the separation of the water from the oil. At high temperature, Yi et al [38] showed that the equilibrium demulsification efficiency with sonochemistry increased with rising temperature. In addition to these areas, more research studies are required in the evaluation of high intensity focused ultrasound, integration of US with more green reagents, development of more comprehensive and enhanced US models in oil technologies and conducting of additional field studies.

### 4.1 High intensity focused ultrasound

**Fig. 12:**
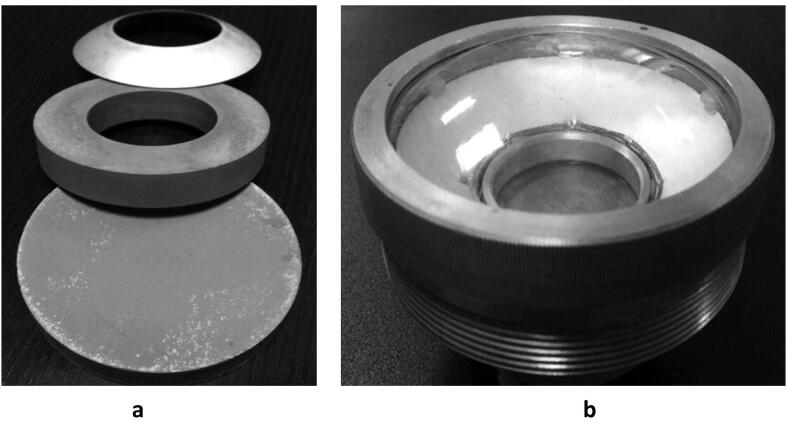
Development of HiFU: **a** The spherical surface [84]**b** Focusing surface showing the poly propylene membrane [84].

The application of high intensity focused ultrasound in oil technologies need to be explored, following the findings from clinical and pharmaceutical research. Currently, the developments in the utilization of high intensity focused ultrasound have been reported in several studies in negligible disruption therapeutic medicine, thrombolysis, hemostasis and drug delivery [84], [85], [86], [87], [88], [89], [90], [91], [92]. This has led to the development of different transducers focusing techniques such as flat, phased array and spherical ultrasonic transducers. In addition, recent progress has been reported on the performance and optimization of HiFU waves in fluids, behavior of interfaces (liquid–air and solid–liquid) under focused US beam and the invasion of blood brain barrier. For example, Shvetsov et al [84] evaluated the characteristics and influence of HiFU waves propagation in aqueous medium through porous piezocomposite transducers. The transducer was spherically focused and has a relative porosity of 18%. The dimensions of the focus material are thickness of 1.2 mm, radius of curvature of 75 mm, aperture of 90 mm and radius of 25 mm (Fig. 12). Paraffin oil was used as immersion fluid, and a resonant frequency of 1.6 MHz was utilized. The resonant frequency was excited at various frequencies through polyethylene or polypropylene membranes. They stated that the acoustic field distribution was significantly affected by changes in the porous piezocomposite element (size and shape) and applied frequency. Dayavansha et al [87] examined the effects of shear waves generated by HiFU on a micellar fluid which consist of sodium salicylate and hexadecyltrimethylammonium bromide at 3:5 ratio and 0.2 M. They reported that the shear waves propagated in a lateral manner from the point of focus of the US. Moreover, phase transition of the micellar fluid was observed at 301 and 311 K.

In an attempt to gain insightful understanding of pulmonary capillary hemorrhage destruction, Patterson and Miller [90] evaluated the acoustic fountain and atomization at liquid–air interface using aqueous and blood surfaces which were stimulated US beam of center frequencies between 5000 and 7200 kHz and mechanical index reaching 1.7. They found that atomization and fountains were present exclusively at mechanical index lower than 1. In their work, Brown et al [91] studied the behavior of HiFU at solid–liquid interface in order to visualize the reflection, scattering and transmission of US under deep tissue ablation conditions. The transducer frequency was set as 670 kHz, visualization was conducted with diffraction-based shadowgraph method and bone (epoxy plate filled with fiber)-water was used as the solid–liquid interface. They noted that destructive interference with the transmitted US tremendously lowered the peak pressure at the beam central focus. Alekou et al [88] investigated the effect of focused US on blood brain barrier destruction through a 3D printed set-up in an attempt to replace animal model experiments. They utilized a transducer frequency of 500 kHz, power of 150 W and pulse of 60 s at 10 ms intervals. They found that the barrier, which was represented as a tube of 40 mm length and 4.4 mm diameter, was ruptured under focused US and 2 mL fluid leakage occurred. These developments should be explored in order to determine their effects on interfaces interactions, formation obstructions and demulsification of crude oil–water mixtures.

### 4.2 US integrated with green reagents as demulsifiers

**Fig. 13:**
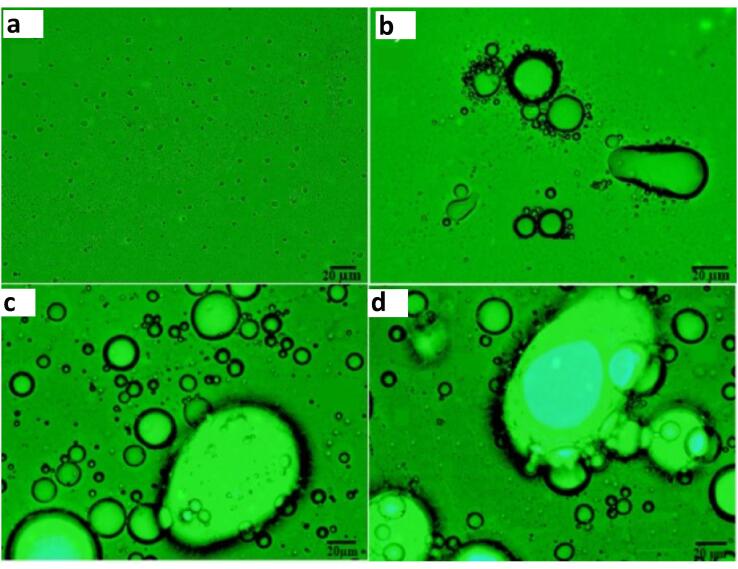
Coalescence using ionic liquids as demulsifier at different times: **a** 0 min **b** 30 min **c** 1 h **d** 2 h [100].

Another area that requires more research studies is in the utilization of US with relatively green demulsifiers and chemical reagents in EOR such as bio-demulsifiers [106], deep eutectic solvents [113], [114], [115] and ionic liquids [98], [99], [100], [101], [102], [103], [104], [105]. There are many studies that have highlighted the environmental benign characteristics of these solvents [93], [94], [95], [96], [97]. In addition to their greenness, they have been described as having low vapor pressure, thermal stability and non-flammability. These properties give them advantages over conventional organic solvents especially under US conditions. Hence, further studies should be conducted to determine the effect of an integrated US and ionic liquid demulsifiers in the dehydration of crude oils. Moreover, the combined effects should be evaluated with standalone US, and the synergistic and behavioral effects of should be established. There are several promising results that have been reported on the application of ionic liquids as demulsifiers, and these could be further enhanced with US. Ionic liquids such as amphiphilic [103], [99], [100], imidazolium [98], [101], [104], [105], pyridinium [104], phosphonium [102] based solvents have been evaluated. In one of such study, Abdullah and Al-Lohedan [99] utilized amphiphilic gemini ionic liquids (GILs) at concentrations between 250 and 1000 ppm for the dehydration of crude oil emulsions with water content of 10, 30 and 50%. The stability of the emulsion, which contain small and uniform water droplets, was confirmed by leaving it for three weeks at 60 °C (Fig. 13). However, with the inclusion of GILs, the water droplets were found to coalesce after 30 min, and the size of the droplets continued to increase as the time progress to 2 h when maximum droplets were noticed.

Although the coalescence enlarged with time, the duration of 2 h is immense and this could reduce productivity and significantly increase the process time. Possible synergistic effects with US should be evaluated as it could hasten the time of separation. Similar studies on the improved tertiary oil recovery should be considered as these solvents have been utilized in flooding agents and surfactants for altering the interfacial properties of emulsions [107], [108], [109], [110], [111], [112], [113], [114], [115].

### 4.3 Detailed and improved model development

Development of more models utilizing US in demulsification and improved oil recovery is crucial as most of the past studies have focused mainly on laboratory scale or field experiments. These models would go a long way in saving cost and process time. Simulation based on techniques such as theoretical modeling with population balance and derived coalescence [40], neural network model [47], phase field coupled with 2D lattice Boltzmann [46] and COMSOL finite element methods [116], [117] have been reported. In one such study, Mohsin and Meribout [116] modeled the improved oil recovery under US application with COMSOL Multiphysics based on finite element method. The model considered acoustic pressure and Darcy flow for the pressure field predictions. In addition, the numerical model utilized a transducer of power of 350 W and frequency of 20 kHz. Crude oil (50 API) and reservoir permeability and porosity of 200 mD and 23%, respectively, was used. The numerical model showed about 70% closeness to the experimental values. The 30% mismatch was attributed to fluctuations in frequency and power which were 0.1 kHz and 20–25 W, respectively. In another study, Mohsin and Meribout [117] simulated the demulsification of water in oil emulsions and observed the US sound pressure level and pressure field using COMSOL. The numerical solution was validated their predictions with experimental results. A frequency of 20 kHz utilized and three different water content scenarios (5, 10, 15%) was modeled. They reported that the pressure field and sound pressure level was similar for the three cases studied. Hence, they inferred that as the water cut increases, more surface area of the droplets is exposed to the pressure field which result in better coagulation. More of these model developments should be investigated as they are essential in optimization and conservation of both resources and time.

### 4.4 Field studies

**Fig. 14:**
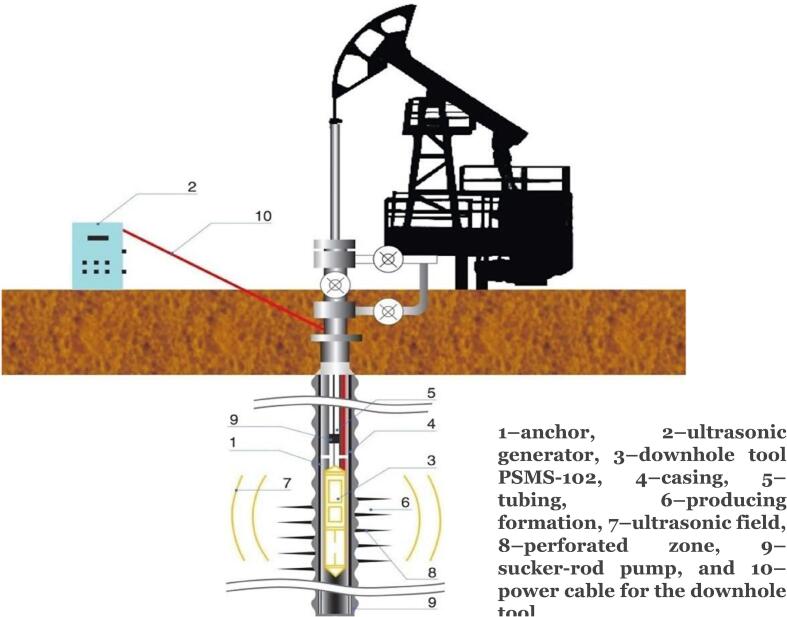
Configuration used in the field study of US stimulation at the Samotlor oil well [120].

There have been developments in field studies on the utilization of US for oil recovery considering their capital intensiveness. However, much of the works in demulsification and improved oil recovery is based on laboratory scale studies. Much of the field studies have been reported by Abrahamov and co-workers [118], [119], [120], [121], [122], [123], [124], [125]. Abramov et al [9] conducted a field study at the Demkinskoe oil production facility using ultrasonic elements in order to determine their effect on viscosity and oil recovery. The oil field initially has a production rate of 1.51 tons day^−1^, water content of 10.3%, production casing of 168 mm, formation pressure of 49 atm, bottomhole pressure of 25.6 atm and temperature of 23 °C. Thereafter, ultrasonic magneto-structure transducer was positioned in the tubing in the perforation region of the downhole. The transducer has a power of 5000 W, frequency of 19 kHz, length of 0.7 m and diameter of 0.102 m. The transducer produced US for 24 h and its element was maintained at temperature lower than 65 °C. They reported that the viscosity of the oil was reduced to 154 mPa s from the initial value of 183 mPa s. Moreover, the oil recovery was enhanced by 26.5% with a production coefficient of 0.094. In a different study, Mullakaev et al [119] the impact of US in the improvement of oil recovery at Samotlor oil field with 27 wells (Fig. 14). The transducer configuration used has similarities to that reported by Abramov et al [9]. However, the transducer was made of piezoceramic element and well logging truck was utilized in placing the cable in the perforated region. The well has a permeability of 0.25 µ m^2^ and water content less than 80%. They reported that the water content of the well fluid was reduced by a mean value of 8.2%. Furthermore, the productivity index and rate of oil production increased by 40% and 75%, respectively. Similar findings of improved oil production at 9 wells of Samotlor field was found by Mullakaev [120] under sono-chemical conditions. The productivity index improved by 107% and oil production rate increased by 5.2 tons day^−1^.

**Fig. 15:**
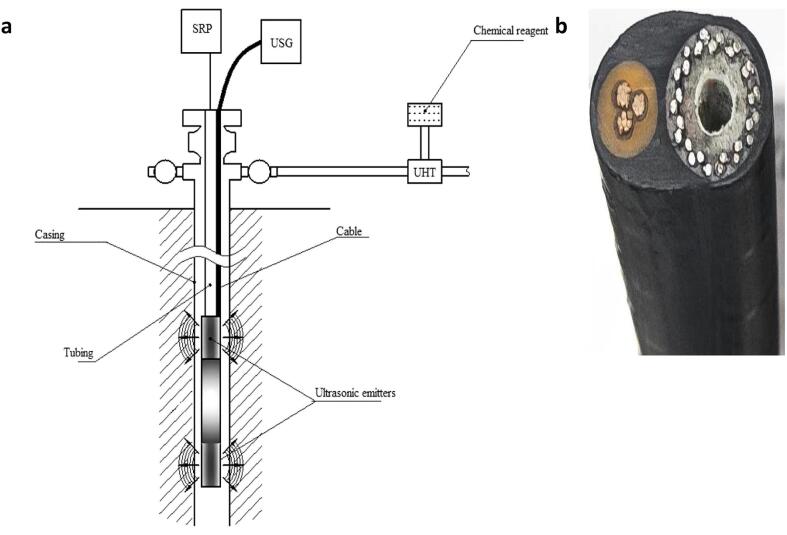
**a** Potential design for commercial application of sonochemical methods [9]**b** Two-in-one cable for transducer and demulsifier delivery downhole [9].

Based on the progress of their field studies, Abramov [9] proposed a configuration that could potentially sustain the viscosity reduction attained by the US in the well bore. They suggested that an extra component for ultrasonic hydrodynamic treatment, in addition to chemicals, for this purpose (Fig. 15). They further proposed a cable that could prevent disruption in the well production during chemical injection. The cable consists of armored pathway for chemical injection on its right and three core conductors on the left for powering the ultrasonic transducer in the downhole. These field studies need to be further developed in order to better understand the effect of different technical and geological conditions on the effectiveness of US.

## 5 Conclusions

Significant developments have been made in the demulsification of medium and heavy crude oil emulsions and improved oil recovery under the application of US. Effect of various US parameters and emulsion properties have been investigated. Generally, enhanced US treatment occurred with less frequency, acoustic intensity lower than critical value, increased irradiation time, pulsed US and lower viscosity. Further research needs to be conducted on unique conditions such as Raschig rings impacts, high temperature and salinity as there are reports that have indicate that they affect US performance. For instance, Ronchi et al [39] observed that the usage of Raschig rings in the acoustic chamber enhanced the separation of oil from water. Moreover, metallic rings such as copper and steel were described as having better demulsification performance as compared to organic rings such as polyvinyl chloride and poly propylene. In their study, Sadatshojaie et al [41] examined the dehydration of three medium crude oil (Crude oil 020, 030 and 040) of different salt concentrations using a static pipe with a volume of 100 cm^3^. They observed that the more the salt content in crude oil, the better the separation of the water from the oil. At high temperature, Yi et al [38] showed that the equilibrium demulsification efficiency with sonochemistry increased with rising temperature. In addition to these areas, more research studies are required in the evaluation of high intensity focused ultrasound, integration of US with more green reagents, development of more comprehensive and enhanced US models in oil technologies and conducting of additional field studies. Technologies such as US assisted green demulsification, high intensity focused ultrasound, and potential pathways in field studies were assessed for their feasibilities. It is essential to evaluate these technologies due to the significant accrued benefits in them. The usage of green demulsifiers such as deep eutectic solvents, ionic liquids and bio-demulsifiers has promising future outlook and US could enhance their technical advancement. HiFU has been applied successfully in clinical research and developments in this area can potentiality improve demulsification and interfacial studies (fluid–fluid and solid–fluid interactions). As regards field studies, there is need to increase actual well investigations because present reports have few on-site measurements with most studies being in laboratory scale. Furthermore, there is need for more detailed modeling of these technologies as it would assist in conserving resources, saving research time and fast-tracking oil production. Additional evaluative studies of conditions such as the usage of Raschig rings, crude oil salinity and high temperature, which have improved demulsification of crude oil emulsions, should be pursued.

## Declaration of Competing Interest

The authors declare that they have no known competing financial interests or personal relationships that could have appeared to influence the work reported in this paper.
